# Histone Purification Combined with High‐Resolution Mass Spectrometry to Examine Histone Post‐Translational Modifications and Histone Variants in *Caenorhabditis elegans*

**DOI:** 10.1002/cpps.114

**Published:** 2020-09-30

**Authors:** Lluís Millan‐Ariño, Zuo‐Fei Yuan, Marlies E. Oomen, Simone Brandenburg, Alexey Chernobrovkin, Jérôme Salignon, Lioba Körner, Roman A. Zubarev, Benjamin A. Garcia, Christian G. Riedel

**Affiliations:** ^1^ Integrated Cardio Metabolic Centre (ICMC), Department of Medicine Karolinska Institute Huddinge Sweden; ^2^ Department of Biosciences and Nutrition Karolinska Institute Huddinge Sweden; ^3^ Department of Biochemistry and Biophysics, Perelman School of Medicine University of Pennsylvania Philadelphia Pennsylvania; ^4^ Epigenetics Institute, Department of Biochemistry and Biophysics, Perelman School of Medicine University of Pennsylvania Philadelphia Pennsylvania; ^5^ European Research Institute for the Biology of Ageing, University Medical Center Groningen (UMCG) University of Groningen Groningen The Netherlands; ^6^ Department of Medical Biochemistry and Biophysics Karolinska Institute Solna Sweden; ^7^ Department of Pharmacological & Technological Chemistry I.M. Sechenov First Moscow State Medical University Moscow Russia

**Keywords:** aging, *Caenorhabditis elegans*, epigenetics, histone post‐translational modifications, histone variants, mass spectrometry

## Abstract

Histones are the major proteinaceous component of chromatin in eukaryotic cells and an important part of the epigenome, affecting most DNA‐related events, including transcription, DNA replication, and chromosome segregation. The properties of histones are greatly influenced by their post‐translational modifications (PTMs), over 200 of which are known today. Given this large number, researchers need sophisticated methods to study histone PTMs comprehensively. In particular, mass spectrometry (MS)−based approaches have gained popularity, allowing for the quantification of dozens of histone PTMs at once. Using these approaches, even the study of co‐occurring PTMs and the discovery of novel PTMs become feasible. The success of MS‐based approaches relies substantially on obtaining pure and well‐preserved histones for analysis, which can be difficult depending on the source material. *Caenorhabditis elegans* has been a popular model organism to study the epigenome, but isolation of pure histones from these animals has been challenging. Here, we address this issue, presenting a method for efficient isolation of pure histone proteins from *C. elegans* at good yield. Further, we describe an MS pipeline optimized for accurate relative quantification of histone PTMs from *C. elegans*. We alkylate and tryptically digest the histones, analyze them by bottom‐up MS, and then evaluate the resulting data by a *C. elegans*−adapted version of the software EpiProfile 2.0. Finally, we show the utility of this pipeline by determining differences in histone PTMs between *C. elegans* strains that age at different rates and thereby achieve very different lifespans. © 2020 The Authors.

**Basic Protocol 1**: Large‐scale growth and harvesting of synchronized *C. elegans*

**Basic Protocol 2**: Nuclear preparation, histone extraction, and histone purification

**Basic Protocol 3**: Bottom‐up mass spectrometry analysis of histone PTMs and histone variants

## INTRODUCTION

**Figure 1:**
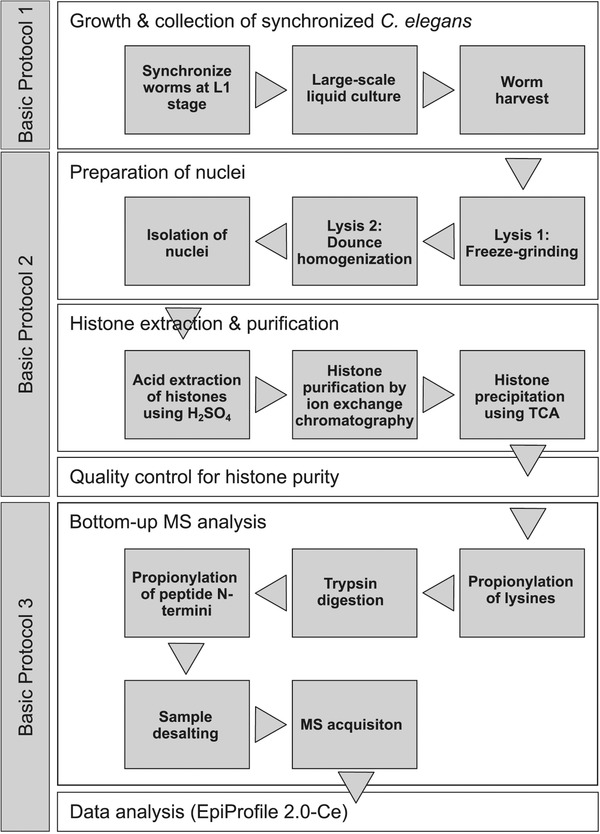
Workflow for the extraction, purification, and mass spectrometric analysis of histone PTMs from *C. elegans*. Synchronized worms are grown on a large scale in liquid culture and eventually harvested (Basic Protocol 1). Upon worm lysis by mechanical methods, nuclei are isolated and histones extracted using acid (H_2_SO_4_). To achieve high histone purity for mass spectrometry, crude histone extracts are subjected to ion‐exchange chromatography. Histone yield and purity are verified by BCA protein quantification and by SDS‐PAGE followed by Coomassie staining (Basic Protocol 2). Finally, histone samples are prepared for bottom‐up mass spectrometry by propionylation and tryptic digestion. After sample desalting, nanoLC‐MS/MS is performed and data analyzed by the software EpiProfile 2.0‐Ce (Basic Protocol 3).

This article provides a detailed set of protocols for the efficient extraction and purification of histone proteins from whole animals of the model organism *Caenorhabditis elegans*, achieving unprecedented purity at sufficient yield for comprehensive analysis of histone post‐translational modifications (PTMs) and histone variants by bottom‐up mass spectrometry. First, we describe how to grow this popular nematode model organism to a large scale by culturing it in liquid medium (Basic Protocol 1). Then, we present a method to isolate and lyse nuclei from the harvested animals, and to eventually purify nuclear histones by cation‐exchange chromatography (Basic Protocol 2). This method achieves sufficient purity so that the obtained histones can be used directly for mass spectrometric analysis, avoiding any additional purification steps (e.g., by SDS‐PAGE or HPLC) that would otherwise lower the yield. Quantification of histone PTMs and histone variants is then achieved by bottom‐up mass spectrometry and subsequent analyses using a *C. elegans*−adapted version of EpiProfile 2.0, a recently developed software to analyze histone PTMs from MS measurements (Yuan et al., 2015, 2018) (Basic Protocol 3). Hereafter, we will refer to our *C. elegans*−adapted version of this software as EpiProfile 2.0‐Ce. Figure 1 depicts a schematic workflow of the procedure in this article, and Tables 1 and 2 summarize all the histone PTMs and their combinations, as well as the histone variants that EpiProfile 2.0‐Ce quantifies. To demonstrate the utility of our method, we determine the histone PTM composition of three *C. elegans* strains that each age at a different rate due to different levels of signaling through the insulin/IGF signaling (IIS) pathway and its downstream transcription factor DAF‐16/FOXO. It has recently been reported that aging is associated with specific changes in histone PTM abundance (Booth & Brunet, 2016; Zhou, Sen, Lin, & Riedel, 2018). Using our method, we identify several expected but also some new histone PTMs whose abundance differs between these three strains.

**Table 1:** Complete List of All the Histone Peptides, Histone Variant Peptides, and Their Modifications Analyzed by the *C. elegans−*adapted Version of EpiProfile 2.0

Histone	Peptide START	Peptide END	Peptide sequence	Peptide modifications analyzed
H3	3	8	TKQTAR	unmod; K4me1; K4me2; K4me3; K4ac
H3	9	17	KSTGGKAPR	unmod; K9me1; K9me2; K9me3; K9ac; K14ac; K9me1K14ac; K9me2K14ac; K9me3K14ac; K9acK14ac
H3	9	17	KSTGGKAPR	S10ph; K9me1S10ph; K9me2S10ph; K9me3S10ph; K9acS10ph; S10phK14ac; K9me1S10phK14ac; K9me2S10phK14ac; K9me3S10phK14ac; K9acS10phK14ac
H3	18	26	KQLATKAAR	unmod; K23me1; K18me1; K18me1K23me1; K18ac; K23ac; K18acK23ac; K23me2; K23me3; K18acK23me1; K18acK23me2; K18acK23me3
H3.3V1	18	26	KALATKAAR	unmod; K23me1; K18me1; K18me1K23me1; K18ac; K23ac; K18acK23ac
H3	27	40	KSAPASGGVKKPHR	unmod; K27me1; K27me2; K27me3; K27ac; K36me1; K27me1K36me1; K27me2K36me1; K27me3K36me1; K27acK36me1; K36me2; K27me1K36me2; K27me2K36me2; K27me3K36me2; K27acK36me2; K36me3; K27me1K36me3; K27me2K36me3; K27me3K36me3; K27acK36me3; K36ac; K27me1K36ac; K27me2K36ac; K27me3K36ac; K27acK36ac
H3.3V1	27	40	KSAIVTGSVKKVHR	unmod; K36me1; K27me1; K27me2; K36me2; K27me3; K36me3; K27me2K36me1; K27me1K36me2; K27me1K36me1; K27me3K36me1; K27me1K36me3; K27me2K36me2; K27me3K36me2; K27ac
H3.3V2	27	40	KSAPTTGGVKKPHR	unmod; K27me1; K27me2; K27me3; K27ac; K36me1; K27me1K36me1; K27me2K36me1; K27me3K36me1; K27acK36me1; K36me2; K27me1K36me2; K27me2K36me2; K27me3K36me2; K27acK36me2; K36me3; K27me1K36me3; K27me2K36me3; K27me3K36me3; K27acK36me3; K36ac; K27me1K36ac; K27me2K36ac; K27me3K36ac; K27acK36ac
H3	54	63	YQKSTELLIR	unmod; K56ac
H3	64	69	KLPFQR	unmod; K64ac
H3	73	83	EIAQDFKTDLR	unmod; K79me1; K79me2; K79me3; K79ac
H3	117	128	VTIMPKDIQLAR	unmod; K122ac
H4	4	17	GKGGKGLGKGGAKR	unmod; K5ac; K8ac; K12ac; K16ac; K5acK8ac; K5acK12ac; K5acK16ac; K8acK12ac; K8acK16ac; K12acK16ac; K5acK8acK12ac; K5acK8acK16ac; K5acK12acK16ac; K8acK12acK16ac; K5acK8acK12acK16ac
H4	20	23	KVLR	unmod; K20me1; K20me2; K20me3; K20ac
H4	24	35	DNIQGITKPAIR	unmod; K31ac
H4	40	45	RGGVKR	unmod; K44me1
H4	68	78	DAVTYCEHAKR	unmod; T71ph
H4	79	92	KTVTAMDVVYALKR	unmod; K79ac
H1	90	107	GVTSKALVQAAGSGANGR	unmod; K94me1; K94ac
H2A	4	18	GKGGKAKTGGKAKSR	unmod; K5ac; K8ac; K10ac; K16ac; K5acK8ac; K5acK10ac; K5acK16ac; K8acK10ac; K8acK16ac; K10acK16ac; K8acK10acK16ac; K5acK10acK16ac; K5acK8acK16ac; K5acK8acK10ac; K5acK8acK10acK16ac
H2AV	1	21	AGGKGKAGKDSGKSKSKVVSR	unmod; K4ac; K9ac; K13ac; K17ac; K4acK9ac; K4acK13ac; K4acK17ac; K9acK13ac; K9acK17ac; K13acK17ac; K9acK13acK17ac; K4acK13acK17ac; K4acK9acK17ac; K4acK9acK13ac; K4acK9acK13acK17ac
H2A	37	43	KGNYAQR	unmod; K37ac
H2A	73	78	DNKKTR	unmod; K75ac
H2B	31	40	KESYSVYIYR	unmod; K31me1; K31me2; K31me3; K31ac
H2B	77	83	LAHYNKR	unmod; K82ac

**Table 2:** List of Histone Peptides Analyzed by the *C. elegans*‐Adapted Version of EpiProfile 2.0 to Compare Abundance Between Canonical Histones and their Variantsa*a*

Histone	Peptide sequence	Coding gene	Histone variant	Description
H3	YRPGTVALR	several	H3	Consensus sequence for canonical histone H3
	VTIMPKDIQLAR	several	H3	Consensus sequence for canonical histone H3
	IRGER	several	H3	Consensus sequence for canonical histone H3
	FRPGTVALR	his‐69, his‐74	H3.3V	Ortholog of human H3.3B
	VTIMPKDMQLAR	his‐69, his‐74, his‐72	H3.3V	Ortholog of human H3.3B
H2A	AGLQFPVGR	several	H2A	Consensus sequence for canonical histone H2A
	HLQLAVR	several	H2A	Consensus sequence for canonical histone H2A
	FLKQR	htz‐1	H2AV	Ortholog of human H2A.Z
	HLHLAIR	htz‐1	H2AV	Ortholog of human H2A.Z
H1	QALKR	his‐24	H1.0	Ortholog of human H1.0

a *Histone peptides analyzed by EpiProfile 2.0‐Ce can be further customized according to user's need to analyze other histone variants than those presented here as an example*.

## Basic Protocol 1 LARGE‐SCALE GROWTH AND HARVESTING OF SYNCHRONIZED *C. ELEGANS*

Our protocol has been optimized to obtain histones of high purity and sufficient yield for mass spectrometry analyses while preserving the histones’ post‐translational modifications (PTMs). Since the histone content in relation to body weight is rather low in *C. elegans* and since some loss occurs throughout the protocol, a substantial amount of starting material is needed. Thus, large quantities of animals need to be grown, which is most easily achieved using liquid culture.

The actual required amount of animals will depend on the purpose of the experiment. Normally, 0.5−1 million animals are sufficient to study most of the histone PTMs in adult wild‐type animals. However, if one would like to detect particularly rare PTMs or analyze a *C. elegans* mutant that bears lower histone content, a higher starting amount is required. Here, it is important to know that in adult *C. elegans*, the tissue contributing the most to histones is the germ line. Thus, any mutant that lacks or has an impaired germline may need to be grown on an increased scale to ultimately yield the same histone amount as wild‐type animals.

### Materials

Desired worm strains, grown on 100‐mm‐diameter Nematode Growth Medium (NGM) plates (see recipe), e.g.:
  Wild‐type *C. elegans* strain (N2)
  Slow‐aging *C. elegans* strain with a hypomorphic insulin/IGF receptor allele (*daf‐2(e1370ts)III*) (CB1370)
  Fast‐aging *C. elegans* strain with a null allele for DAF‐16/FOXO (*daf‐16(mu86lf)I*) (CF1038)
150‐mm NGM plates (see recipe)
50× OP50‐1: Stationary OP50‐1 *E.coli* culture (obtained from the CGC), 50‐fold concentrated relative to an overnight LB culture, and resuspended in S‐Basal (see recipe)
M9 buffer (see recipe)
Hypochlorite solution (see recipe)
1 M MgSO_4_ stock solution
S‐Complete medium (see recipe)
5‐fluoro‐2′‐deoxyuridine (FUDR; Sigma‐Aldrich, F0503)
Sterile Milli‐Q water or equivalent
Nuclear Isolation Buffer (NIB; see recipe)
200 mM AEBSF (4‐(2‐aminoethyl) bezenesulfonyl fluoride hydrochloride; Sigma‐Aldrich, A8456)
2.5 µM microcystin (Sigma‐Aldrich, M4194)
5 M sodium butyrate (Sigma‐Aldrich, B5887)
0.9 M dithiothreitol (DTT; ThermoFisher Scientific, R0861)
100× cOmplete^TM^ EDTA‐free Protease Inhibitor Cocktail (Roche, 11873580001)
0.1 M spermine (Sigma‐Aldrich, S3256)
0.1 M spermidine (Sigma‐Aldrich, 05292)
Liquid nitrogen

15‐ml conical centrifuge tubes
Bench‐top centrifuge for 15‐, 50‐, and 500‐ml tubes (e.g., ThermoFisher Scientific MegaFuge 40R)
Vortex (e.g., Vortex Genie 2)
Stereomicroscope (e.g., Olympus SZX7)
Rotary mixer
2‐L Erlenmeyer flasks
Spectrophotometer (e.g., ThermoFisher Scientific NanoDrop 2000c) and disposable 1‐ml cuvettes
Shaking incubator (e.g., New Brunswick Innova 44R)
500‐ml conical centrifuge tubes (Corning, 431123)
40‐µm cell strainers (if needed, e.g., BD Falcon, 352340)
50‐ml conical centrifuge tubes

### Egg preparation to synchronize C. elegans animals at the L1 larval stage

1 For each sample/condition, start with five 100‐mm‐diameter NGM plates that are crowded with unsynchronized animals. Transfer these animals to ten 150‐mm‐diameter NGM plates each seeded with 1 ml of 50× OP50‐1 *E. coli*. Incubate plates at the desired temperature until the plates are crowded with gravid adults. Typically, the growth temperature for wild‐type animals is 20°C. However, depending on the strain used (e.g., when it carries a temperature‐sensitive allele), other temperatures in the range from 15°C to 25°C may be preferred.
  In order to prevent worms from digging into the agar, which would cause starvation and thereby affect the animals’ physiology, avoid incubating them for extended periods of time on 150‐mm diameter plates (2 days at maximum).
2 Wash worms off the plates using 12 ml of M9 buffer.
3 Collect worms in a 15‐ml tube and centrifuge 1 min at 1500 × *g* to pellet the worms. Discard the supernatant.
4 Repeat this harvesting/washing step until all worms have been removed from the plate and the supernatant from step 3 is clear (approximately three washes).
5 After the final wash, resuspend worms in 7.5 ml sterile M9 buffer.
  If your worm pellet is bigger than 1 ml, split the sample into multiple tubes and add M9 to 7.5 ml final volume, to bring the pellet volume below 1 ml.
6 Add 2.5 ml of fresh hypochlorite solution.
7 Vortex tube for 30 s.
8 Examine under a stereomicroscope to determine if most worm corpses have disappeared and many released eggs can be seen.
9 Repeat steps 7 and 8 until all eggs have been released and barely any corpses remain. Overall, this bleaching (steps 7 to 9) will take approximately 6 min.
10 Centrifuge the tube 1 min at 1500 × *g* to pellet the released eggs.
11 Carefully take off the supernatant and discard it.
12 Wash eggs at least five times, each time with 10 ml M9 buffer, centrifuging 1 min at 1500 × *g* between washes.
  Make sure to go through the first two washes quickly, as a toxic amount of bleach will still remain during the first washes.
13 Spin down eggs 1 min at 1500 × *g*, and resuspend eggs in 10 ml M9 buffer supplemented with 1 mM MgSO_4_.
14 Rotate the tube containing the eggs for 1 day at room temperature or 2 days at 15°C.

### Large‐scale growth of C. elegans in liquid culture

15 Check that there are no unhatched eggs left, and determine the concentration of L1s (first stage larvae) by placing three spots of 5 or 10 µl of the *C. elegans* suspension on an NGM plate and counting the L1 larvae under a stereomicroscope.
16 For each sample/condition, prepare a 2‐L Erlenmeyer flask containing 1 L of S‐Complete Medium.
17 Seed the 1 L of S‐Complete Medium with ∼80 ml of 50× OP50‐1 bacteria.
  Prior to setting up the liquid culture, make sure to prepare enough 50× OP50‐1. You may also have to grow more OP50‐1 during the experiment, to refeed the animals. 50× OP50‐1 can be stored for a few days at 4°C.
18 Check the OD at 600 nm (OD_600_), e.g., using a spectrophotometer. The OD should be between 2 and 4. For accurate results, use disposable cuvettes, use S‐Complete Medium as blank, and dilute your culture 1:10.
19 Inoculate the culture with synchronized L1s from the hatched egg preparation. The L1 concentration should not exceed 1000 animals/ml. (e.g., for 1 L of culture, inoculate with 0.5−1 million L1s).
20 Incubate worm culture in a shaking incubator at the desired temperature and 140 rpm. Typically, the growth temperature for wild‐type animals is 20°C. However, depending on the strain used (e.g., when it carries a temperature‐sensitive allele), other temperatures in the range from 15°C to 25°C may be preferred.
21 Check the OD_600_ of the culture daily and keep it between 2 and 4 A. Add 50× OP50‐1 bacteria when needed.
22 Keep worms in culture until the desired developmental or adult stage. Up to that point, replace S‐Complete Medium every 2‐3 days with fresh medium and new bacteria. This is done by transferring the culture into 500‐ml conical centrifuge tubes, allowing the worms to settle by gravity for ∼10 min, removing the supernatant, and replacing it with new S‐Complete Medium and 50× OP50‐1 *E. coli* as in steps 16‐18.
  Try to maintain the same worm/bacteria/medium ratio throughout the whole experiment, to have stable and reproducible growth conditions.
  If you would like to analyze adult stages beyond day 1 of adulthood, the production of progeny will interfere with the experiment, leading to overcrowding of the culture with animals from the next generation. To avoid this, conditionally sterile C. elegans mutants (e.g., the strain CF512) can be used, or a sterilizing drug can be added prior to progeny production [e.g., 50 µM 5‐fluoro‐2′‐deoxyuridine (FUDR)].

### Harvest worms at a defined time point

From this point on, use cold buffers and cold centrifuges, and work on ice or in a cold room.

23 Transfer the culture into 500‐ml conical centrifuge tubes.
  If the worm culture contains undesired eggs or worms in early larval stages, 40‐µm cell strainers can be used to remove them by filtering the culture before proceeding to the next steps.
24 Allow the worms to settle for 10 min on ice, then centrifuge 3 min at 1500 × *g*, 4°C. Discard the supernatant.
  If you only have a limited number of 500‐ml conical centrifuge tubes available, you can harvest more than 500 ml of culture in the same tube by repeating steps 23‐24.
25 Transfer the worms to 50‐ml centrifuge tubes.
26 Wash the worms with M9 buffer and collect them by spinning them down 2 min at 1500 × *g*, 4°C. Repeat this wash step until the supernatant is cleared of bacteria (approximately three washes).
27 Transfer the worms to 15‐ml tubes.
28 Wash the worms once with 10 ml Milli‐Q water, then centrifuge 2 min at 1500 × *g*, 4°C.
29 Take off supernatant to just above the worm pellet.
30 Mix one volume of worm pellet with two volumes of ice‐cold NIB^+^ buffer.
  To make NIB^+^ buffer, freshly supplement Nuclear Isolation Buffer (NIB) with protease inhibitors and stabilizing agents:
  500 µM AEBSF
  5 nM microcystin
  10 mM sodium butyrate
  1 mM DTT
  1× cOmplete protease inhibitor cocktail
  0.15 mM spermine
  0.15 mM spermidine.
31 Write down the total volume of the worm suspension (worm pellet plus NIB^+^ buffer; used later in Basic Protocol 2) and then snap‐freeze the worms by slowly dripping the suspension into a bucket filled with liquid nitrogen (to make beads).
  PAUSE POINT: Frozen worm beads can be stored at −80°C for the long term.

## Basic Protocol 2 NUCLEAR PREPARATION, HISTONE EXTRACTION, AND HISTONE PURIFICATION

The following acid extraction protocol is optimized to universally isolate all histone proteins and their variants. However, for the more efficient extraction of certain histone variants with unique biochemical and biophysical properties, other methods might be considered. For instance, extraction by diluted perchloric acid is a powerful tool to specifically extract more unstructured histone H1 variants (Zougman & Wiśniewski, 2006).

After acid extraction, we achieve histone purification and enrichment by subjecting the extracted material to ion‐exchange chromatography. Histone proteins are rich in basic amino acid residues, and are thus positively charged. In vivo, this promotes their interaction with the negatively charged phosphate backbone of DNA. In vitro, we can make use of this property. Due to their positive charge, histones have a high affinity for cation‐exchange resins, which allows for their efficient separation from other contaminants (see Rodriguez‐Collazo, Leuba, & Zlatanova, 2009).

### Materials

Frozen worm beads in NIB^+^ buffer (Basic Protocol 1, step 31)
Nuclear Isolation Buffer (NIB; see recipe)
200 mM AEBSF (4‐(2‐aminoethyl) bezenesulfonyl fluoride hydrochloride; Sigma‐Aldrich, A8456)
2.5 µM microcystin (Sigma‐Aldrich, M4194)
5 M sodium butyrate (Sigma‐Aldrich, B5887)
0.9 M dithiothreitol (DTT; ThermoFisher Scientific, R0861)
100× cOmplete^TM^ EDTA‐free Protease Inhibitor Cocktail (Roche, 11873580001)
0.1 M spermine (Sigma‐Aldrich, S3256)
0.1 M spermidine (Sigma‐Aldrich, 05292)
2× Buffer H (see recipe)
5 M NaCl
B‐Hypo‐Lys Buffer (see recipe)
0.4 N H_2_SO_4_ (see recipe)
SP Sepharose High Performance resin (GE Healthcare, 17‐1087‐01)
Equilibration buffer (see recipe)
1 M Tris·Cl, pH 8.0 (Current Protocols, 1998)
0.5 M EDTA, pH 8.0 (Current Protocols, 1998)
Histone wash buffer (see recipe)
Histone elution buffer (see recipe)
Trichloroacetic acid (TCA)
Acetone
50 mM NaHCO_3_
Pierce^TM^ BCA Protein Assay (ThermoFisher Scientific, 23225; also see Current Protocols article: Gallagher, 2012)
1 M Tris base
Milli‐Q water or equivalent
2× Laemmli sample buffer (see Current Protocols article: Gallagher, 2012)
4%‐20% pre‐cast Tris‐Glycine gels (e.g., ThermoFisher Scientific, EC6026BOX)
Tris‐Glycine SDS (TGS) running buffer (e.g., BioRad, 1610732)
Coomassie solution (see recipe)
Destaining solution (see recipe)

Cryogenic grinder: e.g., CryoMill (Retsch)
Stereomicroscope (e.g., Olympus SZX7)
15‐ml Dounce homogenizer set (Sigma‐Aldrich, D9938)
7‐ml Dounce homogenizer set (Sigma‐Aldrich, D9063)
15‐ml centrifuge tubes
Benchtop refrigerated centrifuge for 15‐ and 50‐ml tubes (e.g., ThermoFisher Scientific MegaFuge 40R)
Benchtop refrigerated microcentrifuge (e.g., Eppendorf 5424R)
Large refrigerated centrifuge with rotor for round‐bottom tubes (e.g., Beckman Avanti with JA‐15.50 rotor)
13‐ml round‐bottom centrifuge tubes with lid (Sarstedt, 55.518 and 65.816)
Rotary mixer
1.5‐ml microcentrifuge tubes
Poly‐Prep chromatography columns (Bio‐Rad, 7311550)
pH test paper
50‐ml centrifuge tubes

Additional reagents and equipment for protein assay (see Current Protocols article: Olson & Markwell, 2007) and gel electrophoresis (see Current Protocols article: Gallagher, 2012)

### Freeze‐grinding and nuclear preparation

**Figure 2:**
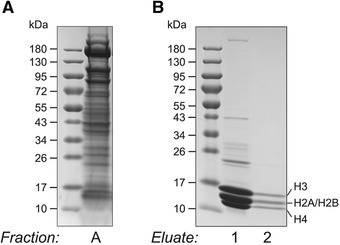
SDS‐PAGE and Coomassie staining of crude *C. elegans* lysate and purified histones. (**A**) Starting material for the histone purification (Basic Protocol 2). Wild‐type whole worms after mechanical lysis (sample A in the protocol). (**B**) The purified histones that were obtained after the completion of Basic Protocol 2. Two consecutive fractions of the histone eluate after ion‐exchange chromatography and histone precipitation (eluate 1 and eluate 2 in the protocol). These eluates look sufficiently clean and are ready to be analyzed by nanoLC‐MS/MS.

1 To fragment the frozen worms, freeze‐grind the worm beads from Basic Protocol 1 using a cryogenic grinder. Using this device, grind until the worms are entirely destroyed and no worm fragments remain visible upon checking a small amount of the ground material under a stereomicroscope after thawing.
  For us, using a CryoMill (Retsch), this takes five consecutive cycles of 3 min at 25 Hz followed by 2 min cooling at 5 Hz. Those settings are only a suggestion and were optimized for 0.5−1 million adult wild‐type animals. They might need further adjustment depending on the starting amount of C. elegans, their developmental or adult stage, and their genotype.
  PAUSE POINT: Collect all powder from the grinder and transfer it immediately to −80°C, where it can be stored long term.
  IMPORTANT NOTE: Perform all subsequent steps on ice at 4°C to avoid enzymatic activities that could alter histone PTMs.
2 Add four volumes of NIB^+^ buffer relative to the total volume of worm suspension in Basic Protocol 1, step 31.
  To make NIB^+^ buffer, freshly supplement Nuclear Isolation Buffer (NIB) with protease inhibitors and stabilizing agents:
  500 µM AEBSF
  5 nM microcystin
  10 mM sodium butyrate
  1 mM DTT
  1× cOmplete protease inhibitor cocktail
  0.15 mM spermine
  0.15 mM spermidine.
  Thaw on ice.
3 Transfer the sample to a pre‐chilled Dounce homogenizer. Use either a 7‐ml or 15‐ml Dounce homogenizer depending on the sample volume.
4 Continue the lysis of the animals and their cells using 50 strokes in the Dounce homogenizer on ice (10 strokes with pestle A and 40 strokes with pestle B).
  If the sample volume exceeds the capacity supported by the Dounce homogenizer, then conduct the Dounce homogenization in batches.
5 Transfer the sample to a pre‐chilled 15‐ml tube. Split into several tubes if needed.
6 Take 50 µl of the sample and keep it at −80°C for later analysis.
  This is sample A: whole worm extract; see Figure 2A for SDS‐PAGE and Coomassie staining of whole worm extract starting material.

7 Spin the sample for 2 min at 200 × *g*, 4°C, to get rid of any debris.
8 Transfer the supernatant to a pre‐chilled 13‐ml round‐bottom tube. Split into several tubes if needed. Keep pellets at −80°C for later optional analysis.
  This is sample B: debris pellet.
9 Collect the nuclei by centrifuging the 13‐ml round‐bottom tube 10 min at 10,000 × *g*, 4°C.
10 Remove the supernatant containing the cytoplasmic fraction and store it at −80°C for later optional analysis.
  This is sample C: cytoplasm.
11 Disrupt the pellet containing the intact nuclei by resuspending in 1.67 ml of Buffer H^+^ per 1 ml of total volume of worm suspension that was snap‐frozen (see last step of Basic Protocol 1).
  To prepare Buffer H^+^, freshly supplement Buffer H with:
  400 mM NaCl
  500 µM AEBSF
  5 nM microcystin
  10 mM sodium butyrate
  2 mM DTT
  1× cOmplete protease inhibitor cocktail.
12 Transfer homogenate to a pre‐chilled Dounce homogenizer and break the nuclei (10 strokes with pestle A, and 40 strokes with pestle B). Use either a 7‐ml or 15‐ml Dounce homogenizer depending on the sample volume.
  If the sample volume exceeds the capacity supported by the Dounce homogenizer, then conduct the Dounce homogenization in batches.
13 Transfer the homogenate to a 13‐ml round‐bottom tube and rotate it for 30 min at 4°C on a rotary mixer.
14 Spin the sample for 10 min at 10,000 × *g*, 4°C, to pellet the chromatin.
15 Collect the supernatant and store it at −80°C for later optional analysis.
  This is sample D: nucleoplasm.

### Acid extraction of histones

16 Wash the chromatin pellet three times in 1 to 3 ml B‐Hypo‐Lys Buffer freshly supplemented with inhibitors:
  500 µM AEBSF
  5 nM microcystin
  10 mM sodium butyrate
  1 mM DTT
  1× cOmplete™ protease inhibitor cocktail.
  Mix well until the pellet is dissolved. For easier pellet resuspension, resuspend first with 100 µl and then add the remaining buffer.
17 Spin 10 min at 10,000 × *g*, 4°C.
18 Collect the supernatant and store it at −80°C for later optional analysis.
  This is sample E: chromatin wash.
19 Re‐spin for 1 min and take off any remaining supernatant after the last wash.
20 Resuspend the pellet in 1 ml 0.4 N H_2_SO_4_ and transfer the sample to a 1.5‐ml microcentrifuge tube.
  Mix well until the pellet is dissolved. For easier pellet resuspension, resuspend first with 100 µl and then add the remaining acid.
21 Rotate on a rotary mixer overnight at 4°C.

### Histone purification by cation‐exchange chromatography

#### Column preparation

For your convenience, you can prepare the resin and column already on the day before the histone purification. The prepared column should be stored at 4°C with the top and bottom of the column properly closed to avoid drying of the resin.

22 Prepare a Poly‐Prep chromatography column by placing it in a stand.
23 Using the end of a Pasteur pipette, push the filter disc to the bottom of the column.
24 Close the bottom of the column using a cap.
25 Add 1 ml Milli‐Q water and mark the top water level on the outside of the column. Open the bottom of the column and allow the water to drain.
26 Close the bottom of the column and add 1 ml of SP Sepharose High Performance resin (gently resuspend the resin before pipetting). Keep adding SP Sepharose until the settled resin reaches the 1‐ml mark made on the column.
27 Place a tube or container underneath the column to collect flow‐through.
28 Add 3 ml of Milli‐Q water, open the bottom of the column, let it drain by gravity flow, then close the bottom of the column. Discard the flow‐through.
  Be careful not to disturb the resin bed when adding liquids to the column during this and the following steps.
29 Repeat step 28 two more times for a total of three washes.
30 Add 3 ml of equilibration buffer, open the bottom of the column, let it drain by gravity flow, then close the bottom of the column. Discard the flow‐through.
31 Repeat step 30 two more times for a total of three washes.
32 Close also the top of the column and store it at 4°C until the histone sample is added.

#### Histone purification

Perform all subsequent steps on ice or at 4°C to avoid enzymatic activities that may alter histone PTMs.

33 After the overnight incubation, spin down the acid extract (from step 21) for 5 min at 14,000 × *g*, 4°C.
34 Collect the supernatant in a 15‐ml centrifuge tube. Keep the pellet at −80°C for later optional analysis.
  This is sample F: acid extraction pellet.
35 Also take 50 µl of the supernatant and store at −80°C for later optional analysis.
  Sample G: acid extract.
36 Neutralize the acid extract by adding an equal volume of 1 M Tris·Cl, pH 8.0.
37 Measure the pH by pipetting 5‐10 µl of neutralized extract onto a pH test paper. At this stage, the pH should be between 7 and 8. If the pH remains lower, add some 1 M Tris base to raise the pH.
38 Supplement the extract to a final concentration of:
  200 mM NaCl
  2 mM EDTA, pH 8.0
  1 mM DTT
  500 µM AEBSF
  5 nM microcystin
  10 mM sodium butyrate
  1× cOmplete™ protease inhibitor cocktail.
39 Take 50 µl of the extract and store it at −80°C for later optional analysis.
  This is sample H: unpurified histone extract.
40 Take the caps off the equilibrated SP‐resin column and place it in a 50‐ml tube, fixing the column in place with tape.
41 Spin 3 min at 100 × *g*, 4°C, to completely remove the equilibration buffer.
42 Close the column with the bottom cap and add 500 µl of the extract.
43 Let the SP‐resin settle on ice for 10 min.
  Carefully flick the column with a finger to release trapped air bubbles.
44 Take the bottom cap off again and add the remaining extract.
45 Allow the extract to pass through the column by gravity flow.
  If here or at any later stage the gravity flow rate becomes unbearably low, you can consider centrifuging the column inside a 50‐ml centrifuge tube for 3 min at 100 × g, 4°C, as done in step 41.
46 Keep the flow‐through for later optional analysis.
  This is sample I: 1^st^ column flow‐through.
47 Wash the SP‐resin with at least 10 ml histone wash buffer by gravity flow.
48 Keep flow‐through for later optional analysis.
  This is sample J: 2^nd^ column flow‐through.
49 Add 1 ml histone elution buffer to the column and collect the eluate. Repeat this step two more times and collect each eluate into a different tube (eluate 1, eluate 2, and eluate 3).

#### Histone precipitation

50 Spin down eluates 15 min at 20,000 × *g*, 4°C.
51 Transfer supernatants to fresh tubes. Make sure not to touch any pellets; it is better to leave a bit of the supernatant behind rather than disturb the pellet.
52 Store pellets at −80°C for later optional analysis.
  This is sample K1: 1^st^ eluate pellet.
53 Spin down again 10 min at 20,000 × *g*, 4°C.
54 Transfer supernatants to fresh tubes. Make sure not to touch any pellets; it is better to leave a bit of the supernatant behind rather than disturb the pellet.
55 Store pellets at −80°C for later optional analysis.
  This is sample K2: 2^nd^ eluate pellet.
56 Add 1/3 volume of 100% trichloroacetic acid drop by drop to the supernatants, then invert tube 10 times to precipitate the histones.
57 Incubate on ice for 30 min at 4°C.
58 Spin down for 20 min at 20,000 × *g*, 4°C.
  The histones will form a thin white layer around the bottom and the sides of the tube. Any significant pellet in the bottom of the tube is often caused by contaminating proteins.
59 Remove supernatant and add 500 µl of ice‐cold acetone (kept at −20°C).
60 Invert three times.
61 Spin down 10 min at 20,000 × *g*. 4°C. Discard the supernatant.
62 Repeat this wash step.
63 Take off all supernatant and let the pellet dry at room temperature for 20 min.
64 Resuspend the histone pellet in 50 µl of 50 mM NaHCO_3_.
65 Using a P10 pipette tip, spot some sample onto pH paper. If the pH is below 7‐8, immediately add some 1 M Tris base to increase the pH accordingly.
  PAUSE POINT: Purified histones can be kept at −80°C until later use.

### Quality control of the histone extraction by SDS‐PAGE and Coomassie staining

To assess the purity of the extracted histones before proceeding to MS analysis, samples are subjected to SDS‐PAGE and Coomassie staining. Core histones are low‐molecular‐weight proteins, migrating between ∼11 and 15 kDa. High‐percentage (15%) or gradient (4%‐20%) Tris‐glycine gels are recommended to efficiently resolve them. Starting material, purified histones, and any optional samples collected throughout the purification can be run together on the same gel. See Figure 2B for an example of how the final histone sample purity compares with the original starting material (Fig. 2A).

66 Estimate histone concentration using a BCA or Bradford assay (see Current Protocols article: Olson & Markwell, 2007).
67 Mix approximately 1 µg of the histone extract with 2× Laemmli buffer.
68 Denature the proteins for 5 min at 95°C.
69 Run the sample on a pre‐cast 15% or 4%‐20% pre‐cast Tris‐glycine gel using Tris‐glycine SDS (TGS) running buffer (also see Current Protocols article: Gallagher, 2012).
70 Stain the gel by slowly shaking in Coomassie solution for 1 hr at room temperature.
71 Destain the gel by slowly shaking in destaining solution. Replace the destaining solution every 30 min until the histone bands have become visible and the gel background is fully destained.

## Basic Protocol 3 BOTTOM‐UP MASS SPECTROMETRY ANALYSIS OF HISTONE PTMS AND HISTONE VARIANTS

In this protocol, the histone samples from Basic Protocol 2 are prepared and analyzed by MS. We use a bottom‐up MS approach, where we first cleave the histones by trypsin into small peptides (5‐15 amino acids in length), separate them by nano‐scale reversed‐phase chromatography (nanoLC), and then quantify them by tandem MS (MS/MS) (Karch, Sidoli, & Garcia, 2016; Sidoli, Bhanu, Karch, Wang, & Garcia, 2016a). However, our approach differs from standard protocols by the addition of two alkylation steps. First, we know that trypsin cuts after lysine and arginine residues and that histones are very basic, containing many of these amino acids. As a consequence, trypsin would normally cleave histones into peptides that are too small for optimal MS/MS analysis (Garcia et al., 2007). To address this, we use propionic acid to alkylate the ζ‐amino groups of unmodified or monomethylated lysine residues, which prevents tryptic digestion at those sites, resulting in peptides that are longer and much more suitable. Second, once tryptic digestion is complete, we again use propionic acid, this time to alkylate each peptide's newly generated N‐terminus. This aids the separation of peptides during nanoLC and further improves MS/MS results. To learn more, also about alternative MS approaches to study histone PTMs and variants, please consult Karch et al. (2016).

Once the MS/MS data have been collected, their analysis is challenging, most importantly because histones carry so many PTMs that can occur alone or in combination with others in a given peptide. This results in a large number of possible peptides that need to be distinguished from others. Another challenge is the existence of isobaric peptides that have the same *m/z* and similar elution times. Thus, we have developed a software application called EpiProfile (Yuan et al., 2015, 2018) to overcome these issues and provide a straightforward solution for the analysis of histone MS/MS data. Here, we use a *C. elegans*−adapted version of this software, called EpiProfile 2.0‐Ce.

### Materials

Purified histone samples in 50 mM NaHCO_3_ or Milli‐Q water (Basic Protocol 2, step 64)
100 mM ammonium bicarbonate (NH_4_HCO_3_), pH 8.0
Glacial acetic acid
Propionic anhydride
Isopropanol
Ammonium hydroxide (NH_4_OH)
Trypsin (Promega)
Wash buffer: 0.1% acetic acid in water
Methanol
Elution buffer: 75% (v/v) acetonitrile/5% acetic acid/20% water
nanoLC buffer A: 0.1% (v/v) formic acid in water
nanoLC buffer B: 0.1% (v/v) formic acid in 75% acetonitrile/25% water

Vacuum centrifuge (e.g., Eppendorf Vacufuge)
pH paper
Vortex (e.g., Vortex Genie 2)
Thermal block (e.g., Eppendorf Thermomix)
1.5‐ or 2‐ml microcentrifuge tubes
Home‐made C_18_ columns or stage‐tips
nano C_18_ column (length: 25 cm, silica tip (outer diameter: 360 µm, inner diameter: 75 µm; New Objective)
Orbitrap Fusion Mass Spectrometer (ThermoFisher Scientific)

### Propionylation of lysine residues

1 Use ∼20 µg of purified histones from Basic Protocol 2, dry samples down in a vacuum centrifuge and resuspend them in 25 µl of 50 mM NH_4_HCO_3_, pH 8.0. Adjust sample concentration with concentrated NH_4_HCO_3_ if samples were in Milli‐Q water, or dry samples down in a vacuum centrifuge and resuspend with NH_4_HCO_3_ if samples were in NaHCO_3_.
2 Use a P10 pipette and spot some sample onto pH paper, to ensure that the pH is around 8.0. Otherwise adjust it by adding glacial acetic acid or powdered NH_4_HCO_3_.
3 Make fresh propionylation reagent by combining propionic anhydride and isopropanol in a 1:3 (v/v) ratio.
  CAUTION: Use fume hood when working with propionic anhydride.
4 Add 10 µl of propionylation reagent to the sample and vortex briefly.
5 Check the pH with pH paper. It now should be between 4 and 6. Immediately add 3‐7 µl NH_4_OH to increase the pH to ∼8.
  Never allow the pH to increase above 10, as other residues such as serine can be propionylated at high pH.
6 Incubate samples 15 min at 37°C.
7 Dry down to less than 5 µl in a vacuum centrifuge. This might take several minutes.
8 Repeat steps 1‐7 once again with fresh reagents to ensure that >95% of the reactions are complete.
9 Resuspend samples in 100 µl of 100 mM NH_4_HCO_3_, pH 8.0.

### Tryptic digestion of the propionylated histones

10 Add trypsin in a 1:20 ratio, i.e., 1 µg trypsin per 20 µg histones.
11 Incubate 6 hr to overnight at 37°C.
12 Quench trypsin by freezing at −80°C or by adding 5 µl glacial acetic acid to lower the pH to ∼4.
13 Dry sample down to 5 µl in a vacuum centrifuge.
  PAUSE POINT: Sample can be stored at −80°C.

### Propionylation of the N‐termini of the tryptic peptides

14 Add 15 µl 100 mM NH_4_HCO_3_, pH 8.0, for a final volume of 20 µl and confirm that the pH has remained around 8.
15 Repeat the derivatization described in steps 1‐7, using fresh reagents.
16 Dry sample down to less than 5 µl in a vacuum centrifuge. This might take several minutes.
17 Add 200 µl of wash buffer to the sample. Check that the pH is below 4.0 with pH paper. If needed, adjust the pH by adding glacial acetic acid.
  PAUSE POINT: Sample can be stored at −80°C.

### Sample desalting with C_18_ Stage‐Tip

This step will remove salt from the digested sample, which otherwise may interfere with the nanoLC‐MS/MS analysis.

18 Construct home‐made C_18_ columns or stage‐tips as described in Sidoli et al. (2016a). Briefly, punch a C18 material disk with a P1000 pipette tip previously cut to make the hole larger, push the minidisk out of the P1000 tip with the help of a fused silica capillary, and place it firmly to the bottom of a P100/200 pipette tip. If desalting more than 25 µg of protein, use two C_18_ minidisks in the same P100/P200 tip.
19 Place the column or stage‐tips in a 1.5‐ml or 2‐ml microcentrifuge tube. Use a centrifuge adapter to hold the column or tip in place.
20 Activate the resin by adding 50 µl methanol to the column/tip and spinning 30‐60 s at 500 × *g*.
21 Repeat step 20.
  From here on, work swiftly to avoid drying of the column/tip.
22 Equilibrate the column by adding 200 µl wash buffer and spinning 30‐60 s at 500 × *g*.
23 Discard flow‐through in the collection tube and repeat step 22.
24 Apply sample from step 17 to the column/stage‐tip.
25 Centrifuge 2‐5 min at 200 × *g*, until all the sample has passed through the column.
26 Wash the column with 50 µl wash buffer and centrifuge 30‐60 s at 500 × *g*.
27 Repeat step 26.
28 Place the column into a new 1.5‐ml microcentrifuge tube and elute the sample with 75 µl elution buffer by spinning 2‐5 min at 200 × *g*.
29 Repeat step 28.
30 Dry down the desalted sample to less than 5 µl in a vacuum centrifuge.
  PAUSE POINT: Sample can be stored at −80°C.

### MS acquisition

In this protocol, bottom‐up MS data are acquired through a combination of data‐dependent acquisition (DDA) with targeted MS2 scans to distinguish coeluting isobaric peptides (see Table 3). To eliminate the need for targeting coeluting isobaric peptides, data‐independent acquisition (DIA) methods can be a good alternative to DDA, although they are not recommended for identifying unknown peptides. For details on how to perform DIA consult Karch et al. (2016).

**Table 3:** Mass List of the Targeted Isobaric Peptides*a*

Mass list of targeted isobaric peptides				
Targeted isobaric peptide	*m/z*	*Z*	*t* start (min)	*t* stop (min)
H3K36me3/K27me2K36me1	543.9860	3	20	40
H3.3K36me3/K27me2K36me1	558.6674	3	20	40
H3K9ac/K14ac	528.2988	2	20	40
H3S10phK14ac/S10phK9ac	568.2790	2	25	40
H3K18me1/K23me1	584.8561	2	30	50
H2A 4_18 3ac	862.9863	2	30	50
H2A 4_18 2ac	869.9941	2	30	50
H4 4_17 3ac	754.9308	2	30	50
H2A 4_18 1ac	877.0019	2	30	50
H3K27me1/K36me1	829.4728	2	30	50
H3.3L2K36me3/K27me2K36me1	574.0200	3	30	60
H4 4_17 2ac	761.9386	2	33	50
H3.3K27me1/K36me1	851.4859	2	35	50
H4 4_17 1ac	768.9465	2	35	55
H2AV 1_21 3ac	794.7711	3	35	60
H2AV 1_21 2ac	799.4430	3	40	60
H3K18ac/K23ac	570.8404	2	40	60
H2AV 1_21 1ac	804.1148	3	40	60
H3.3L2K27me1/K36me1	874.5251	2	50	70

a All the isobaric peptides targeted for MS2 are listed, including their *m/z*, their charge state (*z*), and the retention time coordinates (start and end) during which elution of the respective peptide is expected. Retention coordinates were determined empirically in previous test runs using comparable equipment.

31 Prepare nanoLC Buffers A (0.1% formic acid in water) and B (0.1% formic acid in a solution of 75% acetonitrile/25% water).
32 Program a 70‐min HPLC gradient: starting at 4% B, then linearly increasing B to 35% over 60 min, followed by a linear increase to 95% B over 10 min. The flow rate should be 300 nl/min.
33 We use a nano C_18_ column, 25 cm long with a silica tip. The eluate is electro‐sprayed into the mass spectrometer directly via the column's tip.
34 Program the MS acquisition method for DDA combined with targeted scans. Targeted peptides take priority. See Table 3 for a detailed mass list of the targeted isobaric peptides.
35 Resuspend the sample to ∼1 µg/µl in Buffer A.
36 Load 1‐3 µg of sample onto the nanoLC column.
37 Run the nanoLC‐MS/MS method as programmed in steps 31 and 32.
  Histone peptides are very similar to any other tryptic peptides. Hence, your basic MS setup (column, instrument, and instrument settings) should be the same as you would use for other peptide analyses.

### Data analysis using EpiProfile 2.0‐Ce

**Figure 3:**
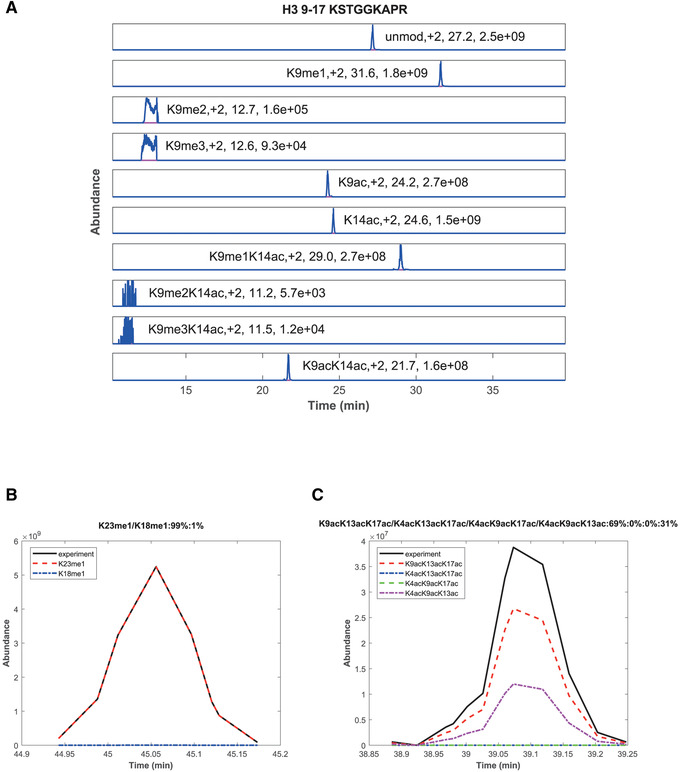
Quantification of histone peptides and discrimination of isobaric peptides. (**A**) Extracted ion chromatograms of the peptide H3 9‐17 in its ten (un)modified forms. The modification state, charge state, retention time, and peak intensity are stated. (**B**) Chromatograms of two isobaric mono‐methylated forms of the H3 18‐26 peptide, H3K18me1 and H3K23me1. They have the same *m/z* ratio and a similar retention time, which makes them indistinguishable when observing their MS1 extracted chromatograms. However, targeted MS2 scans make it possible to distinguish them. (**C**) Analogous to (**B**), chromatograms of four isobaric tri‐acetylated forms of the H2AV 1‐21 peptide are shown, K9acK13acK17ac, K4acK13acK17ac, K4acK9acK17ac, and K4acK9acK13ac. All data presented here were obtained from histones of wild‐type worms.

Although quantification of histone peptides can be achieved manually, e.g., by using the Xcalibur QualBrowser (Thermo) or by using the freeware Skyline (MacLean et al., 2010), we recommend the use of EpiProfile. This software has been specifically developed for the quantification of histone PTMs and variants from MS/MS data, including both DDA and DIA approaches (Yuan et al., 2015, 2018). EpiProfile is a freely available Matlab‐based automated tool that reads raw data files and outputs tables of quantified histone peptides (see Table 4 for an example), layouts (MS1 elution profiles, Fig. 3A), and annotated MS/MS spectra used for identification. EpiProfile is very easy to use and can quantify coeluting isobaric peptides (Fig. 3B and 3C). Since its original release, EpiProfile has been adapted for the study of histones in different organisms including humans, mice, *C. elegans*, and yeast. We recommend consulting previous publications if you would like to obtain a deeper understanding of EpiProfile (Yuan et al., 2015, 2018).

**Table 4:** Example of EpiProfile 2.0‐Ce Quantification Output*a*

Quantification of endogenous histone peptides			
	Wild‐type strain (N2)		
Peptide	RT (min)	Area	Ratio
KSTGGKAPR(H3_9_17)			
Unmod	27.17	1.02E + 11	0.446667
K9me1	31.61	6.59E + 10	0.288417
K9me2	12.67	3.23E + 07	0.000141
K9me3	12.61	2.04E + 07	0.000089
K9ac	24.25	8.89E + 09	0.038923
K14ac	24.61	3.62E + 10	0.158326
K9me1K14ac	29.02	1.48E + 10	0.064777
K9me2K14ac	11.23	3.42E + 04	0.000000
K9me3K14ac	11.46	2.15E + 05	0.000001
K9acK14ac	23.00	4.09E + 09	0.001792

a This is information taken from the EpiProfile Output File histone_ratios.xlsx. The retention time, area under the extracted ion chromatogram (XIC), and relative abundance (ratio) for each (un)modified form of the H3 9‐17 peptide is shown. These data were obtained from histones of wild‐type worms.

## REAGENTS AND SOLUTIONS

### B‐Hypo‐Lys

10 mM HEPES, pH 7.5
1 mM KCl
1.5 mM MgCl_2_
  B‐Hypo‐Lys stock can be stored at 4°C. Just before its use, freshly add additives and inhibitors.

### Buffer H, 2×

50 mM HEPES, pH 7.5
0.2 mM EDTA, pH 8.0
30% (v/v) glycerol
20 mM MgCl_2_
4% (v/v) NP‐40 (Merck, 492016)
  Buffer H (2×) stock can be stored at 4°C. Just before its use, further dilute it to 1× and freshly add additives and inhibitors to obtain Buffer H^+^.

### Coomassie solution

Prepare in Milli‐Q water:
10% (v/v) acetic acid
25% (v/v) 2‐propanol
0.25% (w/v) Coomassie Brilliant Blue R250
Store up to several months at room temperature

### Destaining solution

Prepare in Milli‐Q water:
10% (v/v) acetic acid
10% (v/v) 2‐propanol
Store up to several months at room temperature

### Equilibration buffer

50 mM Tris·Cl, pH 8.0 (Current Protocols, 1998)
2 mM EDTA, pH 8.0
Store up to several months at 4°C

### H_2_SO_4_ , 0.4 N

Bring 2.75 ml of concentrated H_2_SO_4_ to 250 ml with water. Solution can be stored at 4°C.

### Histone elution buffer

50 mM Tris·Cl, pH 8.0 (Current Protocols, 1998)
2 mM EDTA, pH 8.0
2 M NaCl
Store up to several months at 4°C

### Histone wash buffer

50 mM Tris·Cl, pH 8.0 (Current Protocols, 1998)
2 mM EDTA, pH 8.0
0.5 M NaCl
Store up to several months at 4°C

### Hypochlorite solution

1 volume 10 M NaOH
4 volumes 10%‐15% sodium hypochlorite
5 volumes sterile Milli‐Q water
Prepare it fresh before use

### M9 buffer (2 L)

6 g KH_2_PO_4_
12 g Na_2_HPO_4_
10 g NaCl
2 L sterile Milli‐Q water or equivalent
Sterilize by autoclaving
Store up to several months at room temperature

### Nematode growth medium (NGM) plates (high peptone, with streptomycin and Nystatin, 1 L)

3 g NaCl
17 g agar
7.5 g Bacto peptone
975 ml sterile Milli‐Q water or equivalent
Autoclave
After cooling, add the following:
1 ml 5 mg/ml cholesterol in ethanol
1 ml 1 M CaCl_2_
1 ml 1 M MgSO_4_
25 ml 1 M KPO_4_, buffer pH 6.0 (108.3 g KH_2_PO_4_, 35.6 g K_2_HPO_4_, H_2_O to 1 L)
1.25 ml 4400 U/ml Nystatin suspension
2 ml 100 mg/ml streptomycin
Pour into appropriately sized Petri dishes while maintaining sterility
Store up to 1 month at 4°C

### Nuclear isolation buffer (NIB) and NIB^+^buffer

*Prepare NIB stock*:
25 mM HEPES, pH 7.5
0.5 M sucrose
25 mM KCI
10 mM MgCl_2_
0.1 mM EDTA, pH 8.0
Store NIB stock with the above ingredients up to several months at 4°C

### S‐Basal

5.85 g NaCl
1 g K_2_HPO_4_
6 g KH_2_PO_4_
1 ml 5 mg/ml cholesterol in ethanol
Milli‐Q water (or equivalent) to 1 L
Sterilize by autoclaving
Store up to several months at room temperature

### S‐Complete medium

1 L S Basal (see recipe)
10 ml 1 M potassium citrate, pH 6
10 ml trace metals solution (1.86 g disodium EDTA; 0.69 g FeSO_4_•7 H_2_O; 0.2 g MnCl_2_•4 H_2_O; 0.29 g ZnSO_4_ •7 H_2_O; 0.025 g CuSO_4_•5 H_2_O; add Milli‐Q water (or equivalent) to 1 L; sterilize by autoclaving and store in the dark)
3 ml 1 M CaCl_2_
3 ml 1 M MgSO_4_
2 ml 100 mg/ml streptomycin
10 ml 4400 U/ml Nystatin suspension
Mix components while maintaining sterility (do not autoclave)
Prepare it fresh before use

## COMMENTARY

### Background Information

DNA is packaged as chromatin inside the eukaryotic nucleus. The simplest structural unit of chromatin is the nucleosome, composed of ∼147 bp of DNA that wrap around a histone octamer, comprising two copies each of the four core histones H2A, H2B, H3, and H4 (Kornberg, 1974; Luger, Mäder, Richmond, Sargent, & Richmond, 1997). A fifth type of histone, the linker histone H1, sits outside the nucleosome and helps to further stabilize and condense chromatin (Fyodorov, Zhou, Skoultchi, & Bai, 2018; Thoma & Koller, 1977). Chromatin structure and its level of compaction have a direct impact on most DNA‐associated events, including transcriptional regulation, DNA repair, DNA replication, and cell division (Hauer & Gasser, 2017; Hübner, Eckersley‐Maslin, & Spector, 2013; MacAlpine & Almouzni, 2013).

Similar to other proteins in the cell, histones can be post‐translationally modified by different enzymes that deposit or remove chemical groups at individual amino acid residues. Most of these PTMs occur in histones’ exposed N‐ and C‐terminal tails, but some also exist in their globular domains (Kouzarides, 2007). Methylation, acetylation, and phosphorylation are the most common and best‐studied histone PTMs. Nevertheless, there is increasing evidence for other modifications (i.e., ADP‐ribosylation, crotonylation, SUMOylation, or citrullination) that are now studied as well (Zhao & Garcia, 2015). Canonical histones are expressed during S phase of the cell cycle, transcribed from multiple gene copies that are organized in clusters within the genome. During evolution, many histone variants emerged from these canonical histones, all of which have altered biochemical and biophysical properties due to minor sequence differences, and thereby have acquired specific functions needed in specific contexts (Millán‐Ariño, Izquierdo‐Bouldstridge, & Jordan, 2016; Zink & Hake, 2016). In contrast to canonical histones, these variants are typically encoded only from a single gene locus and expressed throughout the cell cycle. Dynamics of both histone PTMs and histone variants are associated with different physiological processes such as cellular differentiation or the aging process (Atlasi & Stunnenberg, 2017; Booth & Brunet, 2016), and their imbalance or dysregulation is tightly linked with many pathologies (Feinberg, Koldobskiy, & Göndör, 2016). This also makes them a potential target to treat such pathologies (Morel, Jeffery, Aspeslagh, Almouzni, & Postel‐Vinay, 2019).

The conventional methods of studying histone PTMs via western blotting or Luminex approaches are limited by the availability of specific antibodies recognizing all these modified residues. Although a broad palette of antibodies is currently available from different vendors, good antibodies are not available for all PTMs, PTM combinations, and variants. Moreover, differences in histone amino acid sequence between species can further limit the availability of good antibodies for less commonly studied organisms. Mass spectrometry methods have recently helped to overcome these issues. They make it possible to study and quantify dozens of histone PTMs in a single experiment, to quantify combinatorial occurrences of PTMs within the same peptides (Karch, DeNizio, Black, & Garcia, 2013), and to obtain this information in any organism. New modifications can even be discovered (Britton, Gonzales‐Cope, Zee, & Garcia, 2011). By now, several studies have described mass spectrometry methods to quantify histone PTMs and variants (Huang, Lin, Garcia, & Zhao, 2015). However, what is crucial for the success of all these approaches is the quality of the histone material to be analyzed. While from some species, e.g., human cells, histones can be obtained rather easily by mere acid extraction, in other species like *C. elegans* this can be very challenging. Particular effort needs to be exercised in order to obtain high‐purity nuclear histone preparations at sufficient yield while preserving their PTMs.

### Critical Parameters and Troubleshooting

#### Synchrony and reproducibility of C. elegans cultures

The methodology described in this article has been developed to study differences in histone PTMs or variant abundance between different *C. elegans* samples. Since the epigenome, and thus these marks, can drastically change depending on environmental influences, including food availability (Berger & Sassone‐Corsi, 2016), or on the developmental stage of the organism (Sidoli, Vandamme, Salcini, & Jensen, 2016b), it is important to avoid any variability in the culturing conditions and to make sure that all samples are perfectly synchronized to the same developmental stage or age. Thus, the protocols provided here for *C. elegans* liquid culture should be strictly followed, and culturing conditions (i.e., temperature and food availability) should be carefully monitored and kept as constant as possible between replicates and throughout the experiment. In particular, we recommend using a temperature tracker, which is placed inside the shaking incubator and checked daily. Furthermore, the abundance of OP50‐1 *E. coli* in the medium is checked daily by OD_600_ measurement, to remain between 2 and 4 absorbance units. For this, 10 ml of culture are transferred to a 15‐ml tube, the *C. elegans* are settled by gravity for 10 min, and then an aliquot of the supernatant is subjected to the OD measurement at 600 nm. Finally, the developmental stage of the animals and thus synchrony between samples and replicates should be checked daily during the first days of the experiment. Should any sample show delays in progressing through development, this delay should be quantified and the sample harvested at an accordingly later time.

#### Input material

The above set of protocols grows 0.5‐1 million *C. elegans* and eventually recovers 20‐30 µg of pure histones. However, sometimes more animals should be used. This is particularly true for certain strains, e.g., strains that have a retarded germline, for the analysis of animals from earlier developmental stages, or when you have an interest in quantifying particularly rare histone PTMs.

#### Achieving optimal yield

Besides increasing the amount of *C. elegans* used, there are a few additional points to consider in order to maximize your histone yield and preserve the histones’ integrity and PTMs. First, it is important to maintain an optimal ratio of biological material and extraction/washing buffers throughout the extraction procedure. Closely follow the ratios indicated in the protocol and scale the buffer amounts depending on the amount of starting material that you use (worm pellet after harvesting). Second, for optimal histone extraction, animals need to be efficiently lysed. In *C. elegans*, this requires breakage of the cuticle, which we achieve by grinding in a cryogenic mill. Grinding results should be checked under a microscope. Upon optimal grinding, all animals should be broken. Ideally, no worm structures should be visible anymore. Further homogenization and lysis is achieved by a Dounce homogenizer. Make sure to use a Dounce homogenizer appropriate for the sample volume and with the correct pestle type.

#### Histone preservation

Proteins and their PTMs are more stable at lower temperatures, when proteases or enzymes that alter their PTMs tend to be less active. Thus, the histone extraction and purification should always be performed on ice or inside a cold room. For similar reasons, we recommend storing all collected samples at −80°C rather than 4°C or −20°C. It is furthermore important to use protease inhibitors and stabilizing agents during the extraction procedure. The choice of stabilizing agents may be adjusted, depending on the histone modifications that you are interested in. In the protocols presented here, microcystin is used to preserve phosphorylations and sodium butyrate is used to preserve acetylations.

#### Ion‐exchange chromatography

One of the main benefits of the protocols described above is the purity of the resulting histone samples. This is achieved by incorporation of an ion‐exchange chromatography step, which removes most non‐histone contaminants. As the purity is so high, no further purification (e.g., gel‐purification or HPLC) is required before trypsin digestion and nanoLC‐MS/MS. However, errors in executing the ion‐exchange chromatography may lead to impurities that interfere with nanoLC‐MS/MS. We therefore recommend always evaluating the purity of your final histone sample by analytical SDS‐PAGE and Coomassie staining. If substantial contamination from non‐histone proteins can be seen, further purification by ion‐exchange chromatography or gel purification should be considered; for future purifications, the ion‐exchange chromatography should be conducted more carefully. Most importantly, make sure to properly neutralize the acid‐extracted samples to a pH of 7‐8 before loading them onto the column.

#### Histone alkylation and digestion

For a good analysis by MS/MS, histones should be optimally propionylated and the tryptic digestion should go to completion. To achieve this, multiple measures should be taken. In the purification protocol's final precipitation step, make sure to properly wash the histone pellet with acetone as described. Any traces of acid that remain with the sample would critically interfere with derivatization and digestion. We also suggest performing the propionylation quickly, not treating too many samples at the same time. And, before initiating the trypsin digestion, carefully confirm that the pH is ∼8.0. Issues with efficient alkylation and digestion would otherwise lead to low detection of the desired peptides.

### Statistical Analysis

**Figure 4:**
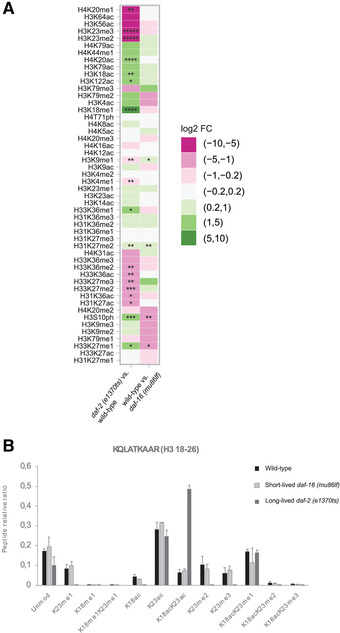
Bottom‐up mass spectrometry uncovers differences in the relative abundance of histone PTMs between wild‐type (N2), long‐lived [*daf‐2(e1370ts)*], and short‐lived [*daf‐16(mu86lf)*] *C. elegans*. (**A**) A heatmap of the changes in H3 and H4 PTM abundances for the comparisons of *daf‐2(e1370ts)* versus wild‐type and wild‐type versus *daf‐16(mu86lf)* animals. Significance of up‐ or downregulated PTMs is indicated by asterisks (* = 0.1 < *p* > 0.05; ** = 0.05 < *p* > 0.01; *** = 0.01 < *p* > 0.001; **** = 0.001 < *p* > 0.0001; ***** = 0.0001 < *p* > 0; *p*‐values were corrected for multiple testing by the False Discovery Rate method). (**B**) Combinatorial histone PTM landscape of the H3 18‐23 peptide in the indicated *C. elegans* strains. Relative ratios of the unmodified and modified forms of the peptide are shown, as calculated by EpiProfile 2.0‐Ce. Error bars represent the standard error across three MS runs.

For the creation of the heat map in Figure 4, the following linear model was used: log2(mark) ∼ date + condition. This allowed us to account for batch effects and to obtain log2 fold‐change values. To account for the high number of tests performed, we corrected our *p*‐values for multiple testing using the False Discovery Rate method. Corrected *p*‐values were computed using the *q*‐value function from the R package *q*‐value. Contrasts were manually set in order to compare different conditions. Clusters for the heatmap were computed by agglomerative hierarchical clustering using the hclust function. All analyses were performed in R version 3.6.3 (R Core Team, 2013) using the packages tidyverse (Wickham, 2016), magrittr (Milton Bache & Wickham, 2014), and Biobase (Huber et al., 2015).

### Understanding Results

The quantification of histone post‐translational modifications in *C. elegans* can be a powerful tool to study how the epigenome contributes to specific aspects of this organism's physiology. To provide an example, we used it to determine, how histone PTMs differ between animals that age either quickly, normally, or slowly.

The nutrient‐sensing insulin/IGF‐like signaling (IIS) pathway is one of the best characterized signaling pathways that regulates the rate at which we age (Kimura, Tissenbaum, Liu, & Ruvkun, 1997; Tatar et al., 2001). When there are plenty of nutrients and IIS is high, aging occurs rather quickly, but when IIS is low, aging is slowed down. Mostly, the latter situation is caused by a transcription factor called DAF‐16/FOXO. This transcription factor is negatively regulated by IIS and drives the expression of many aging‐preventive genes (Murphy et al., 2003; Zhou et al., 2018).

For this study, we chose wild‐type *C. elegans*, which age at a normal rate, a hypomorph of the insulin/IGF‐like receptor *daf‐2* (*daf‐* *2(e1370ts*)), which ages slowly and eventually reaches a very long lifespan, and a null mutant of *daf‐16* (*daf‐16*(*mu86lf*)), which ages quickly and reaches only a very short lifespan. We brought these animals to scale, synchronized them, added 50 µM 5‐fluoro‐2′‐deoxyuridine (FUDR) starting at the L4 stage to prevent progeny production, and eventually grew them until day 2 of adulthood. Animals were then harvested and lysed, and histones extracted, purified, and analyzed by nanoLC‐MS/MS—all according to our protocols above. We then used EpiProfile 2.0‐Ce to analyze the MS/MS data and quantify the histone PTMs and variants. EpiProfile uses previous knowledge about relative elution times for the identification and quantification of histone peptides [for details consult (Yuan et al., 2015, 2018)]. For a given modification on a peptide backbone, EpiProfile calculates its relative abundance or ratio by dividing its area under the MS1 chromatogram by the sum of areas of all (un)modified forms of this same peptide backbone. This information is found in the output file histone_ratios.xlsx (see Table 4 for an example). It allows for the study of all possible modified forms of each peptide, and thus also the study of combinatorial histone PTMs and PTM switches within a given peptide. Besides that, EpiProfile also calculates the relative abundance of individual histone PTMs by summing the relative abundance of all peptides that contain a given modification. These data are found in the output file histone_ratios_single_PTMs.xlsx.

Relative quantification of PTMs on histones H3 and H4 (found in the histone_ratios_single_PTMs.xlsx file) revealed substantial differences in the abundance of certain histone marks in slow‐, normally, and fast‐aging animals. The heat map in Figure 4A illustrates these differences on a log2‐fold scale. Certain marks are positively associated with animals that age more slowly (e.g., H3K18ac or H4K20ac), while others are rather negatively associated (e.g., H3K23me2/3 or H4K20me1). Our data indicate dynamic changes in histone residues H3K18, H3K23, and H4K20, from methylated to acetylated states in animals that age slowly. When looking at the relative abundance of all forms of the H3 18‐26 peptide (found in the histone_ratios.xlsx file), we observed that the increasing acetylation at H3K18 coincides with acetylation of H3K23 of the same peptide (Fig. 4B). We conclude that H4K20ac and likely double‐acetylation of H3 at K18 and K23 are marks associated with slow aging and longevity.

### Time Considerations

The time to grow strains on a large scale before harvesting (Basic Protocol 1) is variable and depends on diverse parameters, e.g., the temperature, the strains used, the desired worm stage to harvest, etc. Whatever the case, the following should give you some guidance. The initial growth of *C. elegans* until they fill multiple 15‐cm plates may take approximately 2 weeks. Egg preparation from gravid adults takes around 2 hr. Hatching of the embryos out of their eggshell and their arrest as L1 larvae is reached after 18 hr at room temperature or 2 days at 15°C. The growth in liquid culture will strongly depend on the stage of animals that you would like to harvest, and can take anywhere from 1 to many days. The harvesting of the liquid culture and freezing of the animals will take about 3 hr.

For Basic Protocol 2, the grinding procedure may take 1 to 2 hr. The resulting worm powder can be stored at −80°C or directly used for nuclear preparation and histone acid extraction. Those steps take 1 full day before leaving samples rotating overnight at 4°C with H_2_SO_4_. Another full day is required for the histone purification by cation‐exchange chromatography and for the histone precipitation step. Quality control of histone samples by SDS‐PAGE and Coomassie staining takes around 4 to 6 hr, including the time it takes to run the protein gels and the time for staining and destaining of the gel.

Finally, sample preparation for nanoLC‐MS/MS and the actual nanoLC‐MS/MS run (Basic Protocol 3) take 2‐3 days. Data analysis by EpiProfile 2.0‐Ce is highly automated and takes only a few minutes to run. However, additional analyses for data representation might take longer.

### Author Contributions

**Lluís Millan‐Ariño**: Data curation; formal analysis; investigation; methodology; writing‐original draft; writing‐review & editing. **Zuo‐Fei Yuan**: Data curation; formal analysis; methodology; software; validation; writing‐review & editing. **Marlies E. Oomen**: Formal analysis; investigation; methodology. **Simone Brandenburg**: Investigation; methodology. **Alexey Chernobrovkin**: Data curation; formal analysis; investigation; methodology. **Jérôme Salignon**: Data curation; formal analysis; investigation. **Lioba Körner**: Investigation; methodology. **Roman A. Zubarev**: Resources; supervision. **Benjamin A. Garcia**: Resources; software; supervision. **Christian G. Riedel**: Conceptualization; funding acquisition; resources; supervision; writing‐original draft; writing‐review & editing.
